# Prevalence of second victims, risk factors and support strategies among young German physicians in internal medicine (SeViD-I survey)

**DOI:** 10.1186/s12995-021-00300-8

**Published:** 2021-03-29

**Authors:** Reinhard Strametz, Peter Koch, Anja Vogelgesang, Amie Burbridge, Hannah Rösner, Miriam Abloescher, Wolfgang Huf, Brigitte Ettl, Matthias Raspe

**Affiliations:** 1 Wiesbaden Business School, RheinMain University of Applied Sciences, Bleichstraße 44, 65183 Wiesbaden, Germany; 2 Centre of Excellence for Epidemiology and Health Services Research for Healthcare Professionals (CVcare), University Medical Centre Hamburg-Eppendorf, 20246 Hamburg, Germany; 3 Department of Cardiology and Pneumology, University Medical Center Göttingen, Robert-Koch-Straße 40, 37075 Göttingen, Germany; 4 Department of Acute Medicine, University Hospitals Coventry and Warwickshire, Clifford Bridge Road, Coventry, CV2 2DX England; 5 Karl Landsteiner Institute for Clinical Risk Management, Wolkersbergenstraße 1, 1130 Vienna, Austria; 6 Clinic Hietzing, Vienna Healthcare Group, Wolkersbergenstraße 1, 1130 Vienna, Austria; 7 Charité – Universitätsmedizin Berlin, Corporate Member of Freie Universität Berlin, Humboldt-Universität zu Berlin, and Berlin Institute of Health, Department of Internal Medicine, Infectious Diseases and Respiratory Medicine, Charitéplatz 1, 10117 Berlin, Germany

**Keywords:** Second victim, Traumatisation, Medical error, Prevalence, Symptoms, Risk factors, Support strategies

## Abstract

**Background:**

Second victims, defined as healthcare team members being traumatised by an unanticipated clinical event or outcome, are frequent in healthcare. Evidence of this phenomenon in Germany, however, is sparse. Recently, we reported the first construction and validation of a German questionnaire. This study aimed to understand this phenomenon better in a sample of young (<= 35 years) German physicians.

**Methods:**

The electronic questionnaire (SeViD-I survey) was administered for 6 weeks to a sample of young physicians in training for internal medicine or a subspecialty. All physicians were members of the German Society of Internal Medicine. The questionnaire had three domains - general experience, symptoms, and support strategies - comprising 46 items. Binary logistic regression models were applied to study the influence of various independent factors on the risk of becoming a second victim, the magnitude of symptoms and the time to self-perceived recovery.

**Results:**

The response rate was 18% (555/3047). 65% of the participants were female, the mean age was 32 years. 59% experienced second victim incidents in their career so far and 35% during the past 12 months. Events with patient harm and unexpected patient deaths or suicides were the most frequent key incidents. 12% of the participants reported that their self-perceived time to full recovery was more than 1 year or have never recovered. Being female was a risk factor for being a second victim (odds ratio (OR) 2.5) and experiencing a high symptom load (OR 2). Working in acute care was promoting a shorter duration to self-perceived recovery (OR 0.5). Support measures with an exceptionally high approval among second victims were the possibility to discuss emotional and ethical issues, prompt debriefing/crisis intervention after the incident and a safe opportunity to contribute insights to prevent similar events in the future.

**Conclusion:**

The second victim phenomenon is frequent among young German physicians in internal medicine. In general, these traumatic events have a potentially high impact on physician health and the care they deliver. A better understanding of second victim traumatisations in Germany and broad implementation of effective support programs are warranted.

## Background

Healthcare is associated with relevant risks not only for patients, but also for healthcare professionals [1]. Besides well-known risks to physical integrity like needle stick injuries [2] or psychological stress [1, 3, 4], unanticipated clinical events or outcomes (often caused by mistakes in healthcare) do not only harm patients. They also traumatise healthcare professionals, who may thus become so-called second victims [5, 6]. Being a second victim may lead to dysfunctional coping strategies [7], resulting in a change in work behaviour and leading to further negative employee-related outcomes. These outcomes are psychological and psychosomatic symptoms [8], including isolation, reduced quality of life up to post-traumatic stress disorder (PTSD) [7, 9, 10], or even suicide [11]. Furthermore, the care of future patients (i.e., practising defensive medicine [12, 13]) can be negatively affected, leading to overall reduced quality of care [14]. Previous surveys in English-speaking countries indicate prevalences up to 42% of second victims among healthcare professionals [15, 16]. Based on the research of the natural history of second victim traumatisation [5], several interventional programs for healthcare professionals were launched, mainly in English-speaking countries [17, 18]. They showed beneficial evidence regarding employee-related outcomes [17, 19], and cost-effectiveness [20].

In Germany, the association of statutory accident insurances defined standards for employees’ care after traumatising events [21]. The first recommendation regarding handling of traumatisations after severe complications in patient care was published in 2013 by the German Society and the German Association of Anaesthesiologists [22]. Contrary to sectors like rail services [23] or air traffic [24], where psychological support for employees after traumatising events has been addressed already, no systematic assessment of this phenomenon in the German-speaking healthcare sector has been published so far.

For this reason, we initiated the SeViD (Second Victims im Deutschsprachigen Raum/Second Victims in German-speaking Countries) project. As a first step, we developed and validated a German-language questionnaire for the assessment of second victim incidents [25].

This study describes the new questionnaire’s first application in a sample of young German physicians, who are in training for general internal medicine or an internal medicine subspecialty. This research was planned and conducted before the SARS-CoV-2 pandemic. Nevertheless, the pandemic is an example, how an unanticipated adverse event can put many healthcare professionals under extreme pressure. Specialists experience high workloads and deal with uncertainty and death while at risk of contracting the illness themselves.

This study aims at adding evidence to the second victim phenomenon by evaluating the following hypotheses:

- The prevalence of second victims among young physicians being trained in internal medicine in Germany is different from reported prevalences from other countries, specialties and age groups.
- Distinct factors can predict the risk of becoming a second victim, the magnitude of symptoms and the time to self-perceived recovery after traumatic events.
- Second victims favour certain support strategies.

## Methods

### Construction and validation of the SeViD questionnaire

The detailed construction and validation of the questionnaire is described in our recent article [25]. Since the original publication is the German language, we provide a brief description: A systematic literature search identified existing questionnaires evaluating the second victim phenomenon in healthcare. Based on these sources (six questionnaires from nine resources), a new German-language questionnaire was developed and tailored to our previously established needs in terms of brevity, straightforward applicability to different groups of healthcare professionals in European healthcare systems and covering broad aspects of the second victims phenomenon (prevalence, symptoms and support strategies). The preliminary version of this questionnaire was subject to cognitive pre-testing in order to ensure content validity. We included healthcare professionals of different professional groups with or without previous second victim experience to participate as volunteers for all pre-tests after informed consent. An independent researcher conducted all cognitive pre-tests. The final questionnaire consists of three domains and 40 items (Table 1). For the symptoms domain, participants answered by a 3-point (strongly pronounced, weakly pronounced, not pronounced) and for the support strategies domain by a 4-point (very helpful, rather helpful, rather not helpful and not helpful) ordinal scale. The options “Don’t know” and “I cannot judge this”, respectively, were also included.

**Table 1:** Domains and items of the SeViD questionnaire

Domain	Item
**General experience with the second victim phenomenon**	knowledge of the term “second victim”
	lifetime prevalence of second victim experience
	12-month prevalence of second victim experience
	type of key incident
	seeking support after key incident
	types of groups that supported the victim after the key incident
	self-perceived time to full recovery after key incident
**Second victim symptoms**^**a**^	fear of social isolation from colleagues
	fear of losing the job
	lethargy
	depressed mood
	concentration problems
	Recall of the situation outside the workplace
	Recall of the situation at the workplace
	aggressive, risky behaviour
	defensive, overprotective behaviour
	psychosomatic reactions (headaches, back pain)
	difficulties sleeping or excessive need to sleep
	use of substances (alcohol/drugs) due to this event
	feeling of shame
	feeling of guilt
	lower self-confidence
	social isolation
	anger against others
	anger against oneself
	desire to get support from others
	desire to work through the incident for deeper understanding
**Second victim support strategies**	immediate time-out to recover
	access to counselling including psychological/psychiatric services
	opportunity to discuss emotional and ethical issues
	obtaining clear information about processes (e.g. root cause analysis, incident reporting)
	formal peer to peer support
	informal emotional support
	prompt debriefing/crisis intervention
	obtaining guidance for continuing clinical duties
	help communicating with patients
	clear guidance about the roles to be expected after the incident
	help to actively participate to work through this incident
	safe opportunity to contribute insights to prevent similar events in future
	opportunity to seek for legal advice after an incident

^a^ Listed in alphabetical order of the German version of the questionnaire

### Design and conduction of the SeViD-I survey

Reporting of this survey is following the checklist for reporting results of internet e-surveys [26]. The survey was conducted using the commercial application SurveyMonkey® (San Mateo, California, US). The electronic survey was embedded in the official homepage of the German Society of Internal Medicine (DGIM e.V.). An invitation with a link for participation was sent by the society itself to all members in training for general internal medicine or an internal medicine subspecialty, being no more than 35 years of age and working in a hospital (*n* = 3047). A reminder was sent after two and 4 weeks and the survey was closed after 6 weeks. The study period ranged from the 20th of August to the 1st of October 2019. Beforehand, the local regulation authority confirmed that no official ethical approval was mandatory. Data collection was completely anonymised with neither tokens or cookies, nor IP addresses stored. The survey was distributed on six panels. The invitation and the reminder for participation included statements on the purpose of the survey, explanation of the term “second victim”, information on responsible investigators (including a contact email address in case of questions) information on the anonymisation process, length of the study, voluntary participation and data protection. The survey was not communicated outside the above-mentioned sample and was only accessible with the provided link. Because of complete anonymisation, a potential multiple participation and spread of the invitation link to others outside the sample could not be controlled. Six items assessing baseline characteristics (gender, age, years in training, specialty status, time in hospital during last 12 months, principal workplace in a hospital during last 12 months) were added to the SeViD questionnaire described above. The survey used adaptive questioning (e.g. questions of the symptoms domain only to participants who had experienced second victim incidents). Each question had to be answered before moving to the next. Participants were able to move back to change their answers. After completion of the survey, participants could voluntarily register via a separate email address for a raffle of incentives (book vouchers and free access to a DGIM training course). All available data were analysed for each question with each number of data points indicated in the results section.

### Preparation and re-coding of variables for statistical analysis

The continuous variables - age and time in specialty training - were categorised (25–30, 31–32 and 33–36 years of age and 1–3, 4–5 and 6–13 years in training, respectively). The item asking for the principal place of work in a hospital was dichotomised into working predominantly in acute care (intensive/ intermediate care and/or emergency department) versus others. Other dichotomisations were performed for the second victim status (before three categories), having experienced support (before three categories) and time to self-perceived full recovery after the key incident (1 month or less vs. more than 1 month). For estimation of the participants’ symptom load, a sum score was calculated based on the answers to the 20 items of this domain. Answers “strongly pronounced” were counted as 1 and “weakly pronounced” as 0.5 (“not at all” and “don’t know” as 0). After this a sum score for each participant was calculated. Based on the median (which was 8.5) this new variable was dichotomised for establishing a low and high symptom load group.

### Statistics

As parametric methods for statistical hypothesis testing, the t-test for independent samples (with 95% confidence interval) was used to compare two groups. Expected and observed distribution patterns were compared using contingency tables and analysed for statistical significance applying the Chi^2^ test. The influence of independent variables (gender, age, years in training, specialty status, workplace, support and symptoms; different combinations of variables for each model) on dependent variables (a second victim status, symptom load and time to self-perceived recovery) were assessed using binary logistic regression models. All statistical analyses were performed with SPSS Statistics Version 26 (IBM, New York).

## Results

### Response rate, non-responder analysis and baseline characteristics

The mean duration of completion for the questionnaire was 5 min and 1 s. From 3047 invited members of the society 555 took part in this survey. This leads to a response rate of 18% (555/3047). At the end of the first questionnaire domain 21 participants (4%) had left the survey. Four hundred ninety-one participants completed the whole questionnaire (88%). Comparing the target to the study population, more women (59% (1799/3047) vs. 65% (361/555); *p* = 0.01, Chi^2^) and slightly younger participants (mean age ± standard deviation: 31,8 ± 2,2 vs. 31,6 ± 2,2; *p* = 0.04, t-test) took part in this survey. The prevalence of second victims did not vary over the three study periods between invitation and two reminders (*p* = 0.45, Chi^2^). Four participants were 36 years of age (these participants turned 36 after the sample was drawn and before they participated in the survey; these cases were included in the final analyses). The median duration of training was 4 years (mean duration 4.5 ± 1.8 years; *n* = 555). Of physicians being six or more years in training, 52% (68/130) have successfully achieved a first formal specialty degree. In Germany 5 years is the minimum duration to achieve the general internal medicine specialty degree followed by optional additional 3 years for an internal medicine subspecialty; alternatively, an internal medicine subspecialty degree can be obtained directly after a minimum of 6 years. The mean time in patient care during the last 12 months was 10.5 ± 2.9 (median 12) months among the participants (*n* = 541; main reasons for time off are parental leave or research activities). 63% (342/541) predominantly worked on general wards, 24% (128/541) on intermediate or intensive care units, 14% (73/541) in the emergency department, 7% (35/541) performing interventions and other 7% (35/541) working in an outpatient clinic (cumulative percent > 100 because multiple answers were accepted).

### Second victim incidents

**Fig. 1:**
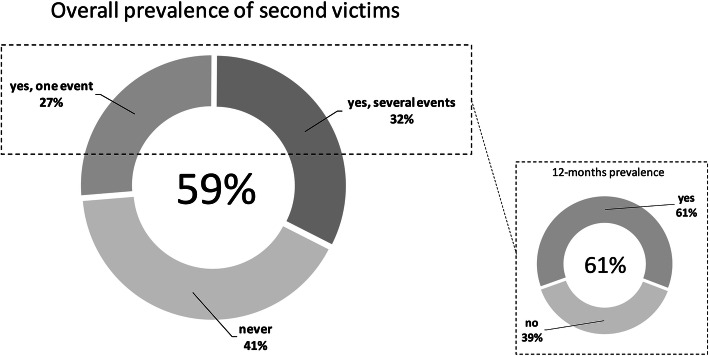
Overall and 12-months prevalence of second victims. Overall prevalence of second victims (*n* = 534): “Never” 41% (220/534), “yes, one event” 27% (141/534) and “yes, several events” 32% (173/534). 12-months prevalence of second victims (*n* = 310): “no” 39% (120/310) and “yes” 61% (190/310)

While 90% (481/534) of the participants had no knowledge of the term “second victim” before invitation to this survey, 59% (314/534) of the participants have experienced a second victim incident before (Fig. 1); from these, 27% (141/534) once and 32% (173/534) several times. 61% (190/310) of second victims experienced at least one incident during the last 12 months. This translates to an overall 12-months prevalence of 35%.

The types of key incidents and self-perceived time until full recovery are displayed in Table 2. Incidents with patient harm (34% (106/310)) and unexpected deaths or suicides of patients (35% (108/310)) were reported as the most frequent key events. Self-perceived time to full recovery after the key event was reported as up to 1 month by 72% (206/287) and as more than 1 month by 28% (81/287) of the participants. 49% (152/310) of the participants received support overcoming the incident from others. 15% (45/310) did not get support, although they asked for it, and other 36% (113/310) did not ask for support. Support came (selection of multiple sources possible) in 82% (117/142) from colleagues, in 60% (84/142) from friends and relatives, in 42% (60/142) from superiors, in 4% (5/142) from professionals (psychiatrists, psychologists) and in 1% (1/142) from the administration.

**Table 2:** Kind of key events and time to self-perceived full recovery among second victims

**Type of key incident**		**Number** (*n* = 310)	**%**
	Event with patient harm	106	34
	Near miss	41	13
	Unexpected death/ suicide of a patient	108	35
	Unexpected death/ suicide of a colleague	5	2
	Aggressive patient or relatives	45	15
	Other types	5	2
**Self-perceived time to full recovery after key incident**		**Number** (*n* = 287)	**%**
	Less than 1 day	13	6
	Within 1 week	94	33
	Within 1 month	99	35
	Within 1 year	47	16
	More than 1 year	9	3
	Never	25	9

### Risks factors for being a second victim

Based on a binary logistic regression model the influence of the independent variables gender, age, years in training, specialty status and working predominantly in acute care on being a second victim was analysed (*n* = 534; Table 3). This model’s risk factor with an odds ratio of 2.5 (95%-CI 1.70–3.55, *p* < 0.01) was female sex. Another risk factor was advanced years in training (6 or more years) with an odds ratio of 2 (95%-CI 1.01–4.23, *p* = 0.046).

**Table 3:** Risk factors for being a second victim

***n*** = 534		Having experienced one/several second victim incidents			
Independent variable		Final model ***r***^**2**^ = 0.09^**a**^			
		ReCoB^**b**^	***p***	odds ratio^**c**^	95%-CI^**d**^
**Gender**^**e**^ (female)		0.90	0.00	2.46	1.70–3.55
**Age** (years)	25–30				
	31–32	0.20	0.41	1.23	0.75–2.01
	33–36	0.15	0.56	1.16	0.71–1.90
**Years in training**	1–3				
	4–5	0.19	0.40	1.21	0.77–1.90
	6–13	0.73	0.05	2.01	1.01–4.23
**Specialty status**^**f**^ (specialist)		−0.09	0.81	0.91	0.42–1.97
**Workplace in acute care**^**g**^		0.25	0.20	1.29	0.88–1.89

For this binary logistic regression model, the dependent variable second victim status was set to never been a second victim vs. having experienced one or several second victim incidents

^a^, Nagelkerkes r^2^; ^b^, regression coefficient B; ^c^, exponentiation of the B coefficient (Exp(B)) or odds ratio; ^d^, confidence interval; ^e^, reference category is male sex; ^f^, reference category is no medical specialty; ^g^, reference category is not working in acute care (predominantly in ICU and/or emergency department)

### Factors with impact on the symptom load of second victims

Based on a binary logistic regression model the influence of the independent variables gender, age, years in training, specialty status and working predominantly in acute care on the symptom load of second victims was analysed (*n* = 314; Table 4). The only risk factor for a high symptom load found in this model with an odds ratio of 2 (95%-CI 1.18–3.36, *p* = 0.01) was female sex.

**Table 4:** Factors influencing the symptom load of second victims

		High symptom load of second victims (*n* = 314)			
Independent variable		Final model ***r***^**2**^ = 0.06^**a**^			
		ReCoB^**b**^	***p***	odds ratio^**c**^	95%-CI^**d**^
**Gender**^**e**^ (female)		0.69	0.01	1.99	1.18–3.36
**Age** (years)	25–30				
	31–32	− 0.22	0.50	0.80	0.42–1.51
	33–36	−0.12	0.72	0.89	0.47–1.70
**Years in training**	1–3				
	4–5	−0.31	0.32	0.74	0.40–1.35
	6–13	−0.58	0.18	0.56	0.24–1.32
**Specialty status**^**f**^ (specialist)		0.69	0.11	2.00	0.85–4.70
**Workplace in acute care**^**g**^		−0.40	0.11	0.67	0.41–1.01

For the construction of the symptom load score, see the Methods section. For this binary logistic regression model, the symptom score was split based on its median in two groups with lower (0 to 8.5 points) vs. higher (9 to 20 points) symptom load scores

^a^, Nagelkerkes *r*^2^; ^b^, regression coefficient B; ^c^, exponentiation of the B coefficient (Exp(B)) or odds ratio; ^d^, confidence interval; ^e^, reference category is male sex; ^f^, reference category is no medical specialty; ^g^, reference category is not working in acute care (predominantly in ICU and/or emergency department)

### Factors with impact on the time to self-perceived full recovery after the key incident

In a third binary logistic regression model the influence of the independent variables gender, age, years in training, specialty status, working predominantly in acute care, having experienced support and a categorised symptom score on the self-perceived time to full recovery (1 month or less versus more than 1 month) of second victims was analysed (*n* = 286; Table 5). The only statistically significantly associated factor was working in acute care with an odds ratio of 0.5 (95%-CI 0.28–0.94, *p* = 0.03), thus promoting a shorter duration to self-perceived full recovery.

**Table 5:** Factors influencing the time to self-perceived full recovery

		Time to full recovery > 1 month (*n* = 286)				
Independent variable		Final model ***r***^**2**^ = 0.10^**a**^				
		ReCoB^**b**^	***p***	odds ratio^**c**^	95%-CI^**d**^	
**Gender**^**e**^ (female)		0.61	0.08	1.84	0.94–3.60	
**Age** (years)	25–30					
	31–32	−0.66	0.08	0.52	0.25–1.10	
	33–36	−0.70	0.06	0.50	0.24–1.04	
**Years in training**	1–3					
	4–5	0.30	0.41	1.35	0.66–2.73	
	6–13	−0.39	0.49	0.68	0.22–2.06	
**Specialty status**^**f**^ (specialist)		0.99	0.07	2.71	0.91–8.10	
**Workplace in acute care**^**g**^		−0.66	0.03	0.52	0.28–0.94	
**Support**^**h**^ (experienced support)		−0.11	0.70	0.90	0.52–1.56	
**Symptoms**^**i**^ (high symptom load)		0.48	0.09	1.62	0.93–2.81	

For this binary logistic regression model the dependent variable time to full recovery was set to up to 1 month vs. more than 1 month

^a^, Nagelkerkes *r*^2^; ^b^, regression coefficient B; ^c^, exponentiation of the B coefficient (Exp(B)) or odds ratio; ^d^, confidence interval; ^e^, reference category is male sex; ^f^, reference category is no medical specialty; ^g^, reference category is not working in acute care (predominantly in ICU and/or emergency department); ^h^, reference category is having experienced no support; ^i^, reference category is a lower symptom load score (further details in the Methods section and Table 4)

### Support strategies for second victims

The participants (*n* = 491) were asked to rate 13 established support strategies for second victims (options were “very helpful”, “rather helpful”, “rather not helpful”, “not helpful” and “I cannot judge this”, Table 6). Support measures rated by > 90% of the second victims as very or rather helpful were the possibility to discuss emotional and ethical issues (93%, 265/287), prompt debriefing/crisis intervention after the incident (95%, 271/287) and a safe opportunity to contribute insights to prevent similar events in the future (92%, 265/287). The ratings of support strategies by second victims versus others were tested for unequal distributions (Chi^2^ tests; 4-point Likert scale). Statistically significant differences were observed for strategy 2 “access to counselling including psychological/psychiatric services” (81% vs. 88% rated rather or very helpful; *p* < 0.01), 11 “help to actively participate to work through this incident” (87% vs. 82% rated rather or very helpful; *p* = 0.02) and 13 “opportunity to seek for legal advice after an incident” (86% vs. 95% rated rather or very helpful; *p* = 0.04).

**Table 6:** Rating of support strategies by participants with and without experience(s) of second victim incidents

Support strategy (*n* = 13)	No second victims (*n* = 204)		Second victims (*n* = 287)		p (Chi^2^)
	Rated rather or very helpful	Rated rather not or not helpful	Rated rather or very helpful	Rated rather not or not helpful	
	% (n)	% (n)	% (n)	% (n)	
**1. Immediate time out to recover**	**69** (140)	**24** (48)	**62** (179)	**34** (97)	0.11
**2. Access to counselling including psychological/psychiatric services**	**88** (180)	**7** (15)	**81** (232)	**15** (44)	< 0.01
**3. Opportunity to discuss emotional and ethical issues**	**92** (188)	**6** (13)	**93** (265)	**6** (16)	0.22
**4. Clear information about processes (e.g. root cause analysis, incident reporting)**	**88** (179)	**10** (20)	**85** (246)	**13** (37)	0.41
**5. Formal peer to peer support**	**78** (160)	**19** (39)	**82** (237)	**15** (43)	0.38
**6. Informal emotional support**	**77** (158)	**17** (35)	**84** (240)	**12** (33)	0.18
**7. Prompt debriefing/crisis intervention**	**91** (186)	**8** (16)	**95** (271)	**4** (10)	0.11
**8. Supportive guidance for continuing clinical duties**	**83** (169)	**13** (26)	**81** (235)	**13** (38)	0.57
**9. Help communicate with patients**	**77** (157)	**18** (37)	**82** (235)	**15** (43)	0.10
**10. Clear guidance about the roles to be expected after the incident**	**71** (144)	**26** (54)	**76** (219)	**16** (47)	0.08
**11. Help to actively participate to work through this incident**	**82** (169)	**12** (24)	**87** (248)	**9** (26)	0.02
**12. Safe opportunity to contribute insights to prevent similar events in future**	**90** (182)	**7** (14)	**92** (265)	**6** (16)	0.72
**13. Opportunity to seek for legal advice after an incident**	**95** (199)	**3** (5)	**86** (249)	**8** (23)	0.04

Assessment of 13 support strategies by 287 s victims and 204 others. For analysis of unequal distribution Chi^2^ tests were applied. Missing % to 100 belong to the option “I cannot judge this”, which is not shown in the table

## Discussion

The survey aimed to investigate the prevalence, influencing factors on occurrence and course as well as support strategies for second victim traumatisations in a cross-sectional fashion among young German physicians working in internal medicine in inpatient care. International studies, especially from the US, suggest [16] that second victim traumatisations are frequent among healthcare professionals and potentially carry a high impact on affected and future patients, the professionals themselves, their colleagues and thus the whole healthcare system. Data from Germany is scarce, which implies a considerable need for more research and campaigns in this country.

Nine out of ten participants of this survey had no knowledge of the term “second victim”. That does not automatically imply that these physicians were unaware of potentially occurring traumatisations at work, but the tendency seems obvious. In contrast, the study of Edrees et al. reported in 2011 that 46% (*n* = 139 participants, manly nurses from Johns Hopkins University/ US) were aware of the term second victim and its definition [27]. One reason could be that healthcare professionals’ traumatisations are until today, to our knowledge, neither mentioned in German medical school or specialty training curricula, nor many support programs exist at German hospitals and medical universities. In 2016, the European Board of Internal Medicine published European standards of postgraduate medical specialist training [28]. Even this modern and comprehensive curriculum addresses traumatisations of healthcare professionals only superficially within the so-called milestones belonging to the CanMEDS framework (e.g. one milestone of the role healthcare advocate “identify, reflect on, and learn from critical incidents such as near misses and preventable medical errors” or one milestone of the role professional “recognise and address personal, psychological, and physical limitations that may affect performance”). Another limitation might be that until today parallel definitions of the term second victims exist (for three frequent definitions, see [16]). In this context, it should be mentioned that the term second victim is criticised by some experts, who argue that it diminishes the importance and seriousness of the injury or complication of the patient and affected relatives [29].

The prevalence of single or multiple second victim traumatisations found in our study among young Germany physicians in internal medicine was high (59% all-over), with 35% of the physicians affected in the last 12 months. A review [16] reported prevalence rates from three studies varying from 10 to 43.3%: A study from Lander et al. from 2006 among otolaryngologists reported a 6-month prevalence of 10% [30], whereas the study of Scott et al. from 2010 among various healthcare professionals including students found a 12-month prevalence of 30% [18] and, finally, the study by Wolf and colleagues from 2000 described the prevalence of 43.1% again among various healthcare professionals [31]. These studies mostly included older populations from different specialties in the United States. Compared to the results of this study, there is no clear signal that prevalences vary substantially with these factors.

Most traumatising incidents from this study where related to situations with direct harm to a patient or even their death. A minor number of cases were near misses or aggressive patients or their relatives. Remarkably, Waterman et al. in a study from 2007 stated that a third of the physicians who “only” have been involved in near misses were suffering from typical second victim traumatisations as well [7].

Most second victims recover soon after traumatising events. Nevertheless, a small but relevant proportion - in our study 12% who need more than 1 year or have not recovered so far - recover late or never. Gazoni et al. report that 19% of traumatised anaesthesiologists have never fully recovered [32]. Especially these colleagues need early and effective help to reduce the risk for severe outcomes like dysfunctional coping strategies, which potentially could harm other patients [7], lead to physical and psychological morbidity [16], or could lead to leaving the profession [19].

Logistic regression models in of our study suggest that women are at greater risk of becoming a second victim (OR 2.5) and having higher symptom loads (OR 2) than men. Besides methodical limitations (e.g. women were overrepresented in the study sample) published studies reported several gender-related differences regarding the second victim phenomenon. Tolin and Foa conclude in a quantitative review from 2006 that females are generally more likely to meet PTSD criteria than men. However, they are less likely to experience potentially traumatic events [33]. Studies by Kaldjian et al. [34], Muller and Ornstein [35] and Wu et al. [36] report more distress among women after traumatising events on one side (e.g. feeling more guilt, being more afraid of losing confidence or reputation), but more constructive patterns of handling the situation compared to men on the other side (e.g. more motivated to discuss errors or to support changes in practice).

In our study, being in advanced training stages (6 years and more) was associated with a higher risk of becoming a second victim (OR 2). In a study by West et al. [15], the prevalence of second victims increased with time from a 3-months prevalence of 14.3% to a 3-years prevalence of 34%. Some authors argue that almost every healthcare professional will experience at least one traumatic event throughout their career [9].

In this study, shorter duration until self-perceived full recovery after a traumatic event was associated with predominantly working in acute care (OR 0.5). An explaining hypothesis could be that adverse events happen more frequently in these fields, so physicians might be better prepared through more routine and expectancy in dealing with such situations.

All regression models show a deficiency in predicting the outcome of the dependent variable. The possible explanation is that the relevant factors have not been included in these models and/or that multiple factors and their complex interactions influence the outcome. Van Gerven et al. lists personal, situational and organisational aspects that impact the outcome [37]. Therefore, further research could concentrate on individual factors like personality characteristics, details of the traumatising events, or environmental conditions to explain differences.

Second victims of this study report that support in overcoming the traumatising event originated mainly from colleagues and friends or relatives, namely from the closest surrounding persons at work and home. Furthermore, second victims ask in particular for support strategies which include a prompt debriefing with discussion of the event and related emotional/ ethical aspects.

Today, nationwide support programs do not exist in the US [5, 7, 38] nor in Europe [39–41]. Single programs have been developed (e.g. in the US: “Medically Induced Trauma Support Service (MITTS)” in Boston [42], the “forYOU” program at the University of Missouri Health Care [18] or the „Resilience in Stressful Events (RISE)” program at Johns Hopkins Hospital [17] and in Europe: “PSUakut”, which is a support program for healthcare professionals working in acute care in Germany [43], the “Mitigating Impact in Second Victims (MISE)” online support program in Spain [40] or “Collegial Help (Kollegiale Hilfe/ KoHi)”, a support program for second victims which is currently established at the Hietzing hospital in Vienna/ Austria [44]). All programs include graduated levels of support. Scott et al. for example describe the following three levels (three tiers): Tier 1 with local unit/department support by direct colleagues, tier 2 with support through trained peer supporters and tier 3 with support through an established referral network (including professional support up to psychologists). The authors estimate that on these levels 60, 30 and 10%, respectively, of all second victims will receive sufficient support. Our study shows that most traumatised physicians who received support get it from their colleagues or friends. According to the just mentioned estimations by Scott et al., up to 40% might not receive the right support they need if professional support programs are not in place.

All support programs for second victims and their prevention aim for strengthening the resilience of healthcare professionals. The term “resilience” has been significantly shaped by the work of Aaron Antonovsky. He defined the sense of coherence as a prerequisite for resilience that is based on three components: viewing the world as comprehensible, meaningful and manageable. Regarding the SARS-CoV-2 pandemic and drawing on current recommendations by Wu et al. we recently published recommendations for healthcare leadership which take these three above-mentioned components into account [45].

Our findings may be limited in several important ways. The cross-sectional design can describe associations but will never link causation. Our sample of young physicians in internal medicine was a convenience sample that is liable to selection bias and thus might lack representativity. More physicians with traumatic incidents in their past could have taken advantage of the survey. More women than expected were among our study participants. Furthermore, investigating only members of one medical society could harbour bias because members could have specific characteristics that might distinguish them from others. Another limitation is the low response rate and the number of dropouts which increased with the duration of the survey (at the last questions around 11%). The fear of potential participants to admit that something went wrong could have negatively influenced the response rate. There is often still a culture of blame in the workplace and fear of recrimination. Furthermore, due to the anonymous conduction of the survey we cannot exclude multiple participations of certain participants. Nevertheless, study characteristics like the response or dropout rates of our electronic survey were among expected limits for such designs. The item “time to full recovery” is difficult to define. Participants might feel that they have fully recovered, but the traumatic incident could still influence their behavior. Additionally, recovery might be a process with ups and downs. Finally, we did not correct for multiple-hypothesis testing. Our analysis is mainly explorative and is supposed to generate hypotheses and a basis for further research in Germany and Europe. Thus, we leave space in drawing the line between statistical significance and clinical relevance to the reader.

## Conclusions

This study describes a high prevalence of second victim traumatisations among young physicians in internal medicine in Germany for the first time. Furthermore, characteristics of these traumatisations have been analysed and support strategies have been evaluated. However, this should be the beginning. There is an obvious need for more research in this field in Germany. It would for example be desirable to understand which environmental conditions and which personality characteristics facilitate traumatisations and lead to worse outcomes. This might help to tailor primary prevention measures and support programs. Establishing nationwide effective support structures for our patients, colleagues and ourselves is our social, ethical and organisational responsibility.

## Data Availability

The datasets analysed during the current study are available from the corresponding author on reasonable request.

## Abbreviations

DGIM: Deutsche Gesellschaft für Innere Medizin (German Society of Internal Medicine)
ICU: Intensive care unit
MISE: Mitigating Impact in Second Victims
MITTS: Medically Induced Trauma Support Service
OR: Odds ratio
PSUakut: Psychosoziale Unterstützung akut (acute psychosocial support)
PTSD: Post-traumatic stress disorder
SARS-CoV-2: Severe acute respiratory syndrome coronavirus type 2
SeViD: Second victims in Deutschland (Germany)
RISE: Resilience in Stressful Events
